# Capecitabine Regulates HSP90AB1 Expression and Induces Apoptosis via Akt/SMARCC1/AP-1/ROS Axis in T Cells

**DOI:** 10.1155/2022/1012509

**Published:** 2022-03-24

**Authors:** Sai Zhang, Shunli Fan, Zhenglu Wang, Wen Hou, Tao Liu, Sei Yoshida, Shuang Yang, Hong Zheng, Zhongyang Shen

**Affiliations:** ^1^School of Medicine, Nankai University, Tianjin, China; ^2^Organ Transplant Department, Tianjin First Central Hospital, School of Medicine, Nankai University, Tianjin, China; ^3^First Central Clinical Institute, Tianjin Medical University, Tianjin, China; ^4^Key Laboratory of Transplant Medicine, Chinese Academy of Medical Sciences, Tianjin First Central Hospital, Tianjin, China; ^5^National Health Commission's Key Laboratory for Critical Care Medicine, Tianjin, China; ^6^Research Institute of Transplant Medicine, Nankai University, Tianjin, China; ^7^Tianjin Key Laboratory for Organ Transplantation, Tianjin, China

## Abstract

Transplant oncology is a newly emerging discipline integrating oncology, transplant medicine, and surgery and has brought malignancy treatment into a new era via transplantation. In this context, obtaining a drug with both immunosuppressive and antitumor effects can take into account the dual needs of preventing both transplant rejection and tumor recurrence in liver transplantation patients with malignancies. Capecitabine (CAP), a classic antitumor drug, has been shown to induce reactive oxygen species (ROS) production and apoptosis in tumor cells. Meanwhile, we have demonstrated that CAP can induce ROS production and apoptosis in T cells to exert immunosuppressive effects, but its underlying molecular mechanism is still unclear. In this study, metronomic doses of CAP were administered to normal mice by gavage, and the spleen was selected for quantitative proteomic and phosphoproteomic analysis. The results showed that CAP significantly reduced the expression of HSP90AB1 and SMARCC1 in the spleen. It was subsequently confirmed that CAP also significantly reduced the expression of HSP90AB1 and SMARCC1 and increased ROS and apoptosis levels in T cells. The results of in vitro experiments showed that HSP90AB1 knockdown resulted in a significant decrease in *p*-Akt, SMARCC1, *p*-c-Fos, and *p*-c-Jun expression levels and a significant increase in ROS and apoptosis levels. HSP90AB1 overexpression significantly inhibited CAP-induced T cell apoptosis by increasing the *p*-Akt, SMARCC1, *p*-c-Fos, and *p*-c-Jun expression levels and reducing the ROS level. In conclusion, HSP90AB1 is a key target of CAP-induced T cell apoptosis via Akt/SMARCC1/AP-1/ROS axis, which provides a novel understanding of CAP-induced T cell apoptosis and lays the experimental foundation for further exploring CAP as an immunosuppressant with antitumor effects to optimize the medication regimen for transplantation patients.

## 1. Introduction

As a prodrug of 5-fluorouracil (5-FU), CAP is converted into 5-FU sequentially by carboxylesterase (CES), cytidine deaminase (CDA), and thymidylate phosphorylase (TP) to exert antitumor effects [1–3]. As a key enzyme for CAP transformation, TP is expressed in many tumor tissues, including colorectal cancer (CRC) and hepatocellular carcinoma (HCC), and it is more concentrated in tumor tissues than in adjacent tissues [4–7]. This distribution is the reason for the significant tumor-targeting capability of CAP. CAP is the first-line therapeutic drug for CRC, and some clinical studies have also confirmed that CAP, especially at the dosage used in metronomic chemotherapy (a novel type of chemotherapy featuring low dosage and uninterrupted administration), has a good effect in treating HCC [8–11]. In addition, the expression of TP in T cells has been confirmed, which lays the pharmacodynamics foundation for the conversion of CAP to 5-FU in T cells [12]. Earlier experiments showed that CAP can induce apoptosis in T cells, which confirmed this view [12]. Therefore, CAP may be a potential immunosuppressant with an antitumor effect. Obtaining an immunosuppressant with an antitumor effect has great clinical application value. This is because, in the context of transplant oncology, although liver transplantation has become an important treatment for HCC and nonresectable colorectal liver metastases, exposure to postoperative immunosuppressive therapy would contribute to increased tumor recurrence and poor outcomes [13–15]. Therefore, a drug with both immunosuppressive and antitumor effects can take into account the dual needs of preventing both transplant rejection and tumor recurrence.

Apoptosis is a kind of programmed cell death, and its role in the antitumor effect of CAP has been confirmed [16, 17]. Induction of T cell apoptosis is one of the classic antirejection mechanisms of immunosuppressants [18–20]. As oxygen-containing chemically reactive molecules, ROS are closely related to T cell apoptosis and activation [21]. On this basis, we explored the immunosuppressive effect of CAP and recently showed that CAP can induce T cell ROS production, subsequently leading to apoptosis, but the molecular mechanism behind it is still unknown [12]. In recent years, the development of mass spectrometry technology has made it possible to identify proteins on a large scale, which enables the underlying mechanism behind the immunosuppressive effect of CAP to be explored [22–24]. So far, there has been no proteomics research on the immunosuppressive effect of CAP. Therefore, in the present study, metronomic chemotherapy doses of CAP were administered to normal mice and quantitative proteomics and phosphoproteomics are applied to look for the target proteins of CAP-induced T cell apoptosis. The results of proteomic analysis showed that CAP significantly reduced HSP90AB1 and SMARCC1 expression. HSP90AB1, which is one of the HSP90 subtypes, has a critical role in tumorigenesis and progression and is also closely related to T cell activity [25–28]. SMARCC1, which is a member of the SWI/SNF DNA chromatin remodeling complex family, is also associated with tumor progression and T cell activity [29, 30]. As previously mentioned, HSP90AB1 can regulate Akt expression, and Akt can regulate the expression of SMARCC1 [26, 31]. Jeong et al. showed that SMARCC1 can reduce the expression of AP-1 to regulate T cell activity [30]. AP-1 is a transcriptional factor that consists of a homodimer or heterodimer of Jun and Fos families [32]. Previous studies have demonstrated that AP-1 can regulate GCLM and HO-1 expression, which in turn influences the production of ROS [33]. ROS production, as previously mentioned, is closely associated with apoptosis [12, 21, 34]. It was hypothesized that targeted inhibition of HSP90AB1 could inhibit Akt/SMARCC1/AP-1 axis to induce ROS production and lead to T cell apoptosis by CAP. In addition, considering the role of HSP90AB1 in tumorigenesis and progression, HSP90AB1 may also be a key target of CAP to exert antitumor effect. In this study, we focused on the underlying mechanism of T cell apoptosis, attempting to confirm that CAP can induce T cell apoptosis via HSP90AB1/Akt/SMARCC1/AP-1/ROS axis.

## 2. Materials and Methods

### 2.1. Animals

All animals were obtained from China National Institutes for Food and Drug Control. Male Balb/c mice aged 6–8 weeks were gavaged with metronomic doses of CAP (100 mg/kg/day) (Solarbio, Beijing, China) [12, 35]. At days 0, 7, 14, and 21, mice (*n* = 10) were sacrificed. All animal experiments followed the ARRIVE guidelines and were approved by the Ethics Committee of Nankai University.

### 2.2. Total Protein Extraction and Protein Quality Test

The spleen sample (*n* = 4/group) was lysed with PASP lysis, and the supernatant was reduced with 10 mM DTT for 1 h after the lysate was centrifuged, and then, alkylated with IAM for 1 h. The samples were mixed with acetone for 2 h, and the precipitation was collected after centrifugation [36]. The protein concentration of the sample was calculated using Bradford protein quantitative kit (Solarbio, Beijing, China).

### 2.3. TMT Labeling of Peptides and Separation of Fractions

A buffer solution of DB dissolution, Trypsin, TEAB, and CaCl2 was added successively to the sample solution. The supernatant was collected and mixed with TMT labeling reagent [37]. The sample was fractionated, and eluates were collected and combined into 10 fractions.

### 2.4. LC-MS/MS Analysis

The Q Exactive™ HF-X mass spectrometer and an EASY-nL C™ 1200 UHPLC system were used to perform Shotgun proteomics analyses. Peptides were separated and analyzed using Q Exactive™ HF-X mass spectrometer.

### 2.5. Data Analysis

Raw data were searched separately against UniPort database. Compared with day 0, the proteins of days 7, 14, and 21, whose quantitation differed significantly (*P* < 0.05 and FC ≥ 1.2 or FC ≤ 0.83), were defined as differentially expressed proteins (DEPs). Next, databases, including ProDom, Pfam, PRINTS, ProSite, SMART, and PANTHER, were used to perform GO and IPR functional analyses. The protein family and pathway analyses were performed using the databases COG and KEGG, and STRING-db server was used to analyze the probable protein–protein interactions.

### 2.6. Sorting Primary CD3^+^ T Cells

The anti-CD3 microbeads (Miltenyi Biotec, Germany) was used to sort CD3^+^ T cells in mouse spleen. Then, anti-CD3 antibody (2 *μ*g/mL) and anti-CD28 antibody (1 *μ*g/mL) (BioLegend, San Diego, CA, USA) were used to activate T cells.

### 2.7. Immunohistochemistry (IHC) Assay

HSP90AB1 and SMARCC1 (Abcam, Cambridge, UK) expression levels in the spleen were determined by IHC assay. The images were acquired using a microscope (400x magnification).

### 2.8. Transfection of siRNA and Plasmid

The sequence of HSP90AB1 siRNA was 5′-GCCCUGGACAAGAUUCGAUTT-3′. The sequence of SMARCC1 siRNA was 5′-GCAGAUGCUCCUACCAAUATT-3′ (GenePharma, Shanghai, China). Lipofectamine 3000 Transfection Reagent (Thermo Fisher, Waltham, MA, USA) was used for siRNA transfection. The HSP90AB1 plasmid was purchased from Genechem Corporation (Shanghai, China), which was also transfected using Lipofectamine 3000 Transfection Reagent.

### 2.9. Apoptosis and ROS Measurement

The apoptosis and ROS detection were performed using an Apoptosis Kit (Solarbio, Beijing, China) and Reactive Oxygen Species Assay Kit (Solarbio, Beijing, China) according to the manufacturers' instructions [12]. The FITC-Annexin V dye and propidium iodide (PI) dye were added successively into the sample, and the proportion of apoptosis was analyzed using flow cytometry. T cells were collected and stained with DCFH-DA. The mean fluorescence intensity (MFI) of DCF was detected using flow cytometry.

### 2.10. Cellular Reduced Glutathione (GSH) Measurement

GSH detection was performed using the GSH Assay Kit (Nanjing Jiancheng Bioengineering Institute, Nanjing, China) according to the manufacturer's instructions [38]. After 5-FU treatment, T cells were fragmented by ultrasound and centrifuged, and the supernatant was collected. Then, GSH standards (20 *μ*mol/L) and working solution were prepared. The sample was mixed with the working solution, and the OD value was measured at 405 nm.

### 2.11. Western Blotting

The expression levels of HSP90AB1, SMARCC1, *p*-Akt, BAX, BCL2, GCLM, GCLC, HO-1 (Abcam, Cambridge, UK), Caspase3 (CST, Massachusetts, USA), *p*-HSP90AB1, *p*-c-Fos, and *p*-c-Jun (ImmunoWay, Texas, USA) were detected. The ImageJ 7.0 software was used to analyze the bands.

### 2.12. Statistical Analysis

The SPSS 13.0 (SPSS GmbH, Munich, Germany) and GraphPad 8.0 software (GraphPad Software, La Jolla, CA, USA) were used to analyze the data. Data were expressed as mean ± standard deviation (SD). One-way analysis of variance (ANOVA) was used to determine the differences between groups, and *P* < 0.05 was considered to be statistically significant.

## 3. Results

### 3.1. Quantitative Proteomic and Phosphoproteomic Analysis Revealed that CAP Inhibits HSP90AB1 and SMARCC1 Protein Expression In Vivo

**Figure 1:**
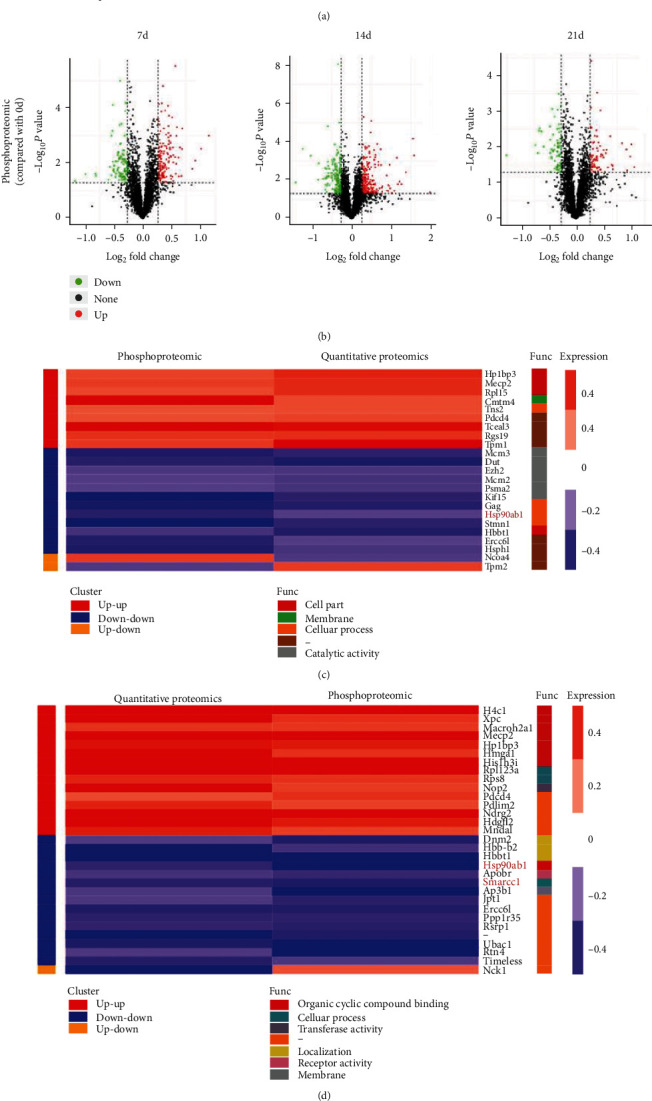
Quantitative proteomic and phosphoproteomic analysis revealed that CAP inhibits HSP90AB1 and SMARCC1 protein expression in vivo. Normal mice were administered metronomic doses of CAP (100 mg/kg/d). On days 0, 7, 14, and 21, the spleen was collected for quantitative proteomic and phosphoproteomic analysis. (a) Volcanic map of differentially expressed proteins (DEPs) in quantitative proteomic analysis on days 7, 14, and 21 (compared with day 0). (b) Volcanic map of DEPs in phosphoproteomic analysis on days 7, 14, and 21 (compared with day 0). Subsequently, association analysis of quantitative proteomic and phosphoproteomic analysis results was performed. (c–e) Heat map of DEPs in both quantitative proteomic and phosphoproteomic analyses on days 7, 14, and 21.

Previous studies have confirmed that CAP can induce T cell apoptosis and has the potential to be an effective immunosuppressant, but the underlying molecular mechanism remains unknown [12]. In the present study, normal mice were gavaged with metronomic chemotherapy doses of CAP, and the immune organ—the spleen—was selected because T cell sorting may have an impact on protein expression, and the sample size cannot meet the needs of proteomic analyses. Thus, direct T cell proteomic testing is difficult. TMT-based quantitative proteomics and phosphoproteomic were used to separately and quantitatively analyze 7,565 proteins and 3398 phosphorylated proteins in the spleen. Quantitative proteomic results (Figure 1(a)) show that compared with day 0, on day 7, 187 proteins were downregulated and 175 proteins were upregulated; on day 14, 257 proteins were downregulated and 244 proteins were upregulated; on day 21, 175 proteins were downregulated and 132 proteins were upregulated. Subsequently, as shown in Supplementary Figure S1, which is a GO enrichment histogram, in the biological process (BP), DEPs are enriched to immune responses, immune system processes, activation of immune response, and so on. In the cellular component (CC), DEPs are enriched to macromolecular complex, intracellular organelles, and so on. In the molecular function (MF), DEPs are enriched to structural molecule activity, structural constituent of ribosome, DNA binding, and so on. Phosphoproteomic results (Figure 1(b)) show that compared with day 0, on day 7, 98 proteins were downregulated and 148 proteins were upregulated; on day 14, 149 proteins were downregulated and 203 proteins were upregulated; on day 21, 63 proteins were downregulated and 76 proteins were upregulated. Subsequently, as shown in Supplementary Figure S2, which is a GO enrichment histogram, in BP, DEPs are mainly enriched to DNA-dependent DNA replication, apoptotic signaling pathway, single-organism transport, and so on. In CC, DEPs are enriched to intracellular nonmembrane-bounded organelles, integral components of membranes, intracellular organelle part, and so on. In MF, DEPs are enriched to ATPase activity, protein serine/threonine kinase activity, enzyme binding, and so on. Because both quantitative proteomic and phosphoproteomic analyses are important methods for studying cell function, in this study, the information from both methods was integrated. As shown in Supplementary Figure S3a, the results of quantitative proteomic analysis and the results of phosphoproteomic analysis were significantly correlated. As shown in Supplementary Figure S3b, compared with day 0, on days 7, 14, and 21, there were 23, 30, and 10 proteins, respectively, with significant changes in both quantitative proteomic and phosphoproteomic analyses. Specific DEPs are presented in Figures 1(c)–1(e), and at each time point, HSP90AB1 and *p*-HSP90AB1 expression in the spleen of mice decreased significantly; on days 14 and 21, both SMARCC1 and *p*-SMARCC1 expression decreased significantly. The results of protein interaction analysis showed that HSP90AB1 and SMARCC1 have the potential for interaction (Supplementary Figure S3c). Studies have confirmed that HSP90AB1 can regulate the expression of Akt, and Akt can regulate the expression of SMARCC1 [26, 31]. The results of protein interaction analysis also showed that HSP90AB1, SMARCC1, and the apoptosis-related proteins have the potential for interaction (Supplementary Figure S3c). Previous studies have confirmed that both HSP90AB1 and SMARCC1 are closely related to T cell activity [25, 30]. Hence, HSP90AB1 and SMARCC1 may be the target protein we searched for, and there could be a possible association between two proteins.

### 3.2. CAP Reduces the Expression of HSP90AB1 and SMARCC1 in the Spleen

**Figure 2:**
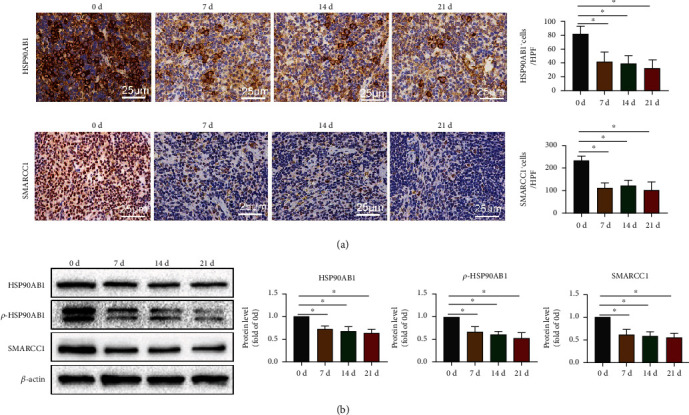
CAP can reduce the expression of HSP90AB1 and SMARCC1 in the spleen of mice. In order to verify the reliability of proteomic results, IHC and western blot were applied to detect HSP90AB1 and SMARCC1 expression in mouse spleen. (a) HSP90AB1 and SMARCC1 in the spleen were stained with IHC (400x). (b) The protein levels of HSP90AB1, *p*-HSP90AB1, and SMARCC1 in the spleen were evaluated using western blot assay. Data are shown as mean ± SD. ^∗^*P* < 0.05.

Next, we utilized western blot and IHC to verify the proteomics results. The IHC results showed that, compared with day 0, the number of HSP90AB1-positive and SMARCC1-positive cells in the spleen of mice was significantly reduced on days 7, 14, and 21 (Figure 2(a)). The western blot results also showed that, compared with day 0, HSP90AB1, *p*-HSP90AB1, and SMARCC1 expression levels were significantly reduced on days 7, 14, and 21 (Figure 2(b)). The above results verify the reliability of the proteomics results and confirm that CAP can reduce HSP90AB1 and SMARCC1 expression in the spleen of mice.

### 3.3. CAP Reduces the Expression of HSP90AB1 and SMARCC1 in T Cells

**Figure 3:**
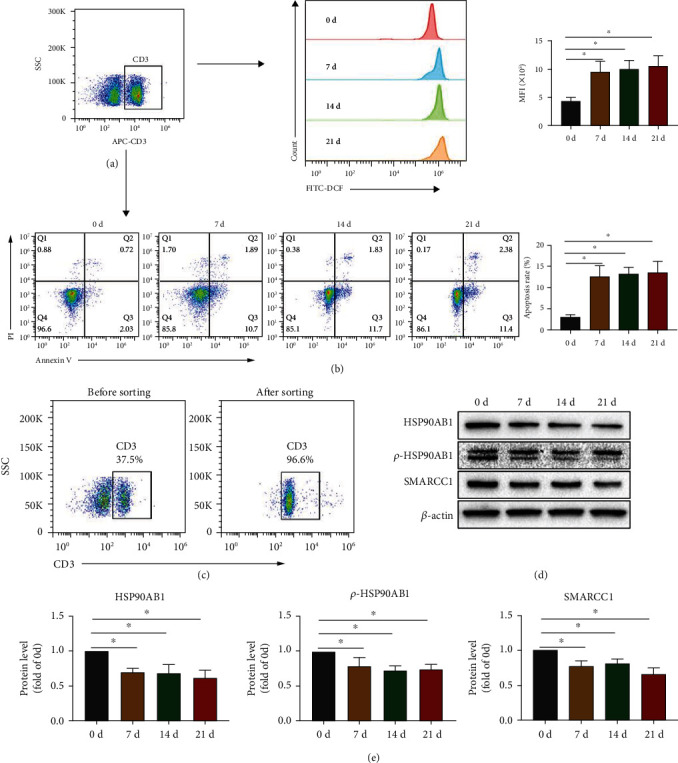
CAP can reduce the expression of HSP90AB1 and SMARCC1 in T cells of mice. Normal mice were gavaged with metronomic doses of CAP (100 mg/kg/d). On days 0, 7, 14, and 21, (a) mononuclear cells extracted from mouse spleen were collected and gated by CD3, and then, the apoptosis rate of CD3^+^ T cells was detected using Annexin V and PI staining. (b) The ROS level of CD3^+^ T cells was evaluated using DCFH-DA staining. (c) CD3^+^ T cells were sorted from mouse spleen and identified by staining with PE-CD3 antibody. (d, e) HSP90AB1, *p*-HSP90AB1, and SMARCC1 expression in T cells was evaluated using western blot assay. Data are shown as mean ± SD. ^∗^*P* < 0.05.

As an immune organ containing T, B, and other types of immune cells [39, 40], there may be inconsistencies between the protein changes in the spleen and the protein changes in T cells. We first confirmed that CAP significantly increased the ROS production and apoptosis rate of T cells, which was consistent with the results of previous studies (Figures 3(a) and 3(b)) [12]. Immediately afterward, CD3^+^ T cells in mouse spleen were sorted using magnetic beads (Figure 3(c)), and western blot analysis confirmed that CAP can significantly reduce the expression of HSP90AB1, *p*-HSP90AB1, and SMARCC1 in T cells (Figures 3(d) and 3(e)).

### 3.4. Knocking Down HSP90AB1 Can Induce T Cell Apoptosis via Akt/SMARCC1/AP-1/ROS Axis

**Figure 4:**
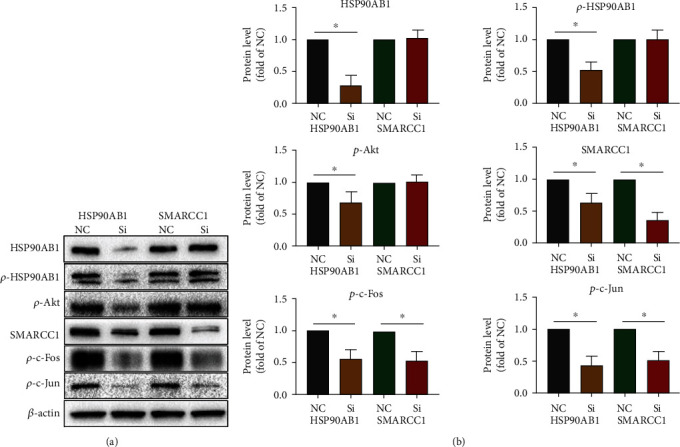
Knocking down of HSP90AB1 can regulate Akt/SMARCC1/AP-1 axis in T cells. Primary T cells were sorted and stimulated in vitro with anti-CD3/CD28 antibodies. Then, HSP90AB1 and SMARCC1 were reduced in T cells by siRNA knockdown. (a, b) The protein levels of HSP90AB1, *p*-HSP90AB1, SMARCC1, *p*-Akt, *p*-c-Fos, and *p*-c-Jun were evaluated using western blot assay. Data are shown as mean ± SD. ^∗^*P* < 0.05. Abbreviations: si: siRNA; NC: negative control.

**Figure 5:**
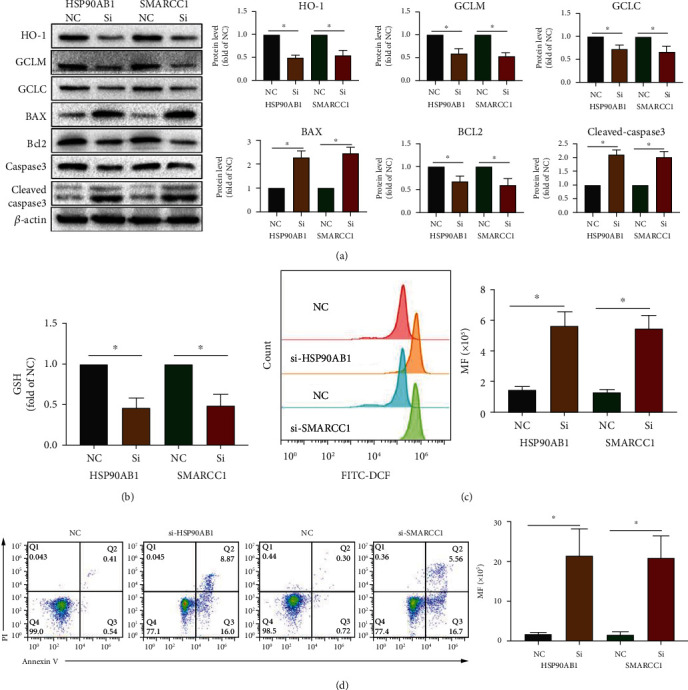
Knocking down of HSP90AB1 and SMARCC1 can repress GCLC, GCLM, and HO-1 expression; reduce GSH level; and increase ROS production and apoptosis rate in T cells. (a) The protein levels of GCLM, GCLC, HO-1, BAX, BCL2, and Caspase3 were evaluated using western blot assay. (b) The GSH level of T cells was evaluated using the reduced GSH Assay Kit. (c) The ROS level of T cells was evaluated using DCFH-DA staining. (d) The apoptosis of T cells was evaluated using Annexin V/PI staining. Data are shown as mean ± SD. ^∗^*P* < 0.05.

As previously mentioned, HSP90AB1 can regulate Akt expression, and Akt can regulate the expression of SMARCC1 [26, 31]. In addition, SMARCC1 can reduce the expression of AP-1 to regulate T cell activity [30]. Previous studies have demonstrated that AP-1 can regulate GCLM and HO-1 expression, which in turn influences the production of ROS [33]. It has been well established that ROS production is closely associated with apoptosis [12, 21, 34]. As shown in Supplementary Figure S3c, the results of protein interaction analysis also showed that HSP90AB1, Akt, SMARCC1, AP-1, HO-1, GCLC, GCLM, and apoptosis-related proteins such as BCL2, BAX, and Caspase3 have the potential for interaction, but this was not confirmed in T cells. Therefore, primary CD3^+^ T cells were obtained by magnetic bead sorting and were activated with CD3 and CD28 antibodies. As shown in Figures 4(a) and 5(a), after knocking down HSP90AB1 in T cells, HSP90AB1, *p*-HSP90AB1, SMARCC1, *p*-Akt, *p*-c-Fos, *p*-c-Jun (both *p*-c-Fos and *p*-c-Jun are subunits of AP-1), HO-1, GCLM, and GCLC expression was significantly reduced; after knocking down SMARCC1 in T cells, no significant changes in HSP90AB1, *p*-HSP90AB1, or *p*-Akt expression were observed, but SMARCC1, *p*-c-Fos, *p*-c-Jun, HO-1, GCLC, and GCLM expression was significantly reduced. Reduced glutathione (GSH) levels (an important biomarker of antioxidant status, which can reduce ROS levels) [33] were lowered significantly (Figure 5(b)), and ROS levels were significantly elevated (Figure 5(c)) after knocking down HSP90AB1 and SMARCC1 in T cells. In turn, knocking down HSP90AB1 or SMARCC1 significantly induced T cell apoptosis (Figure 5(d)). The above results confirm that the HSP90AB1/Akt/SMARCC1/AP-1/ROS axis plays an important role in T cell apoptosis.

### 3.5. Overexpression of HSP90AB1 Can Alleviate CAP-Induced Apoptosis in T Cells via Akt/SMARCC1/AP-1/ROS Axis

**Figure 6:**
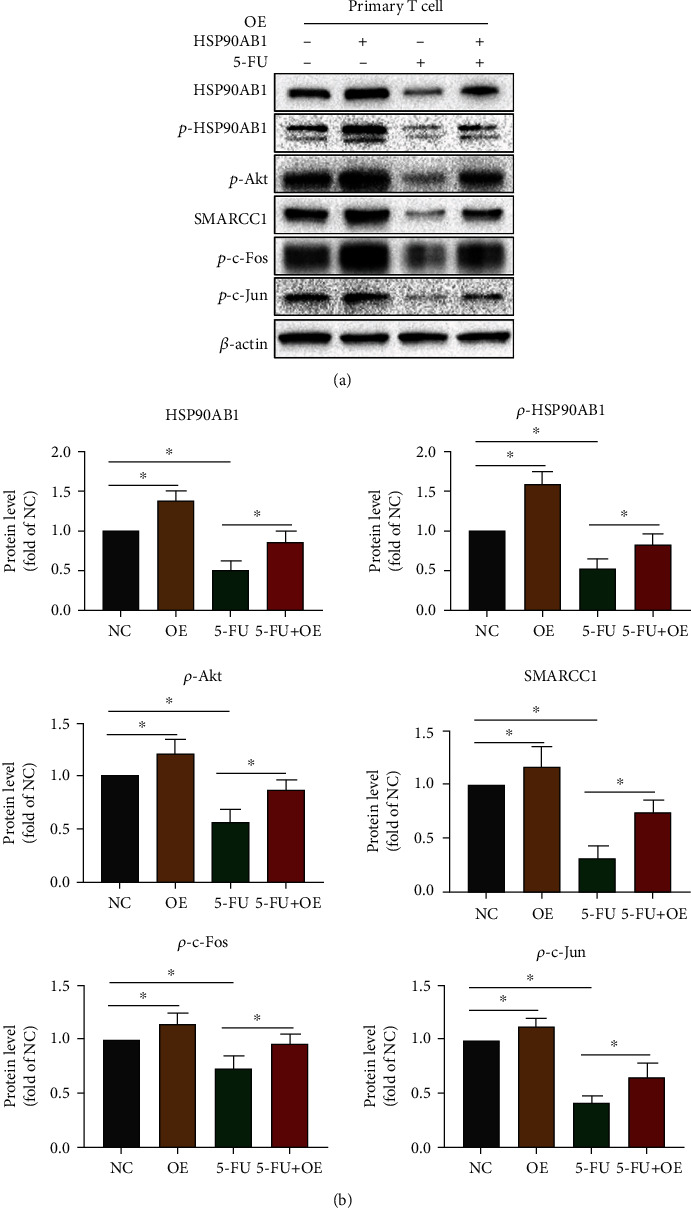
Overexpression of HSP90AB1 attenuated the inhibition of SMARCC1, Akt, and AP-1 expression by CAP in T cells. HSP90AB1 was overexpressed by transfecting with HSP90AB1 overexpression plasmids in primary CD3^+^ T cells. T cells were exposed to 5-FU (the active ingredient of CAP) (0 *μ*M or 10 *μ*M) for 48 h. (a, b) HSP90AB1, *p*-HSP90AB1, SMARCC1, *p*-Akt, *p*-c-Fos, and *p*-c-Jun expression was evaluated using western blot assay. Data are shown as mean ± SD. ^∗^*P* < 0.05. Abbreviation: OE: overexpression.

**Figure 7:**
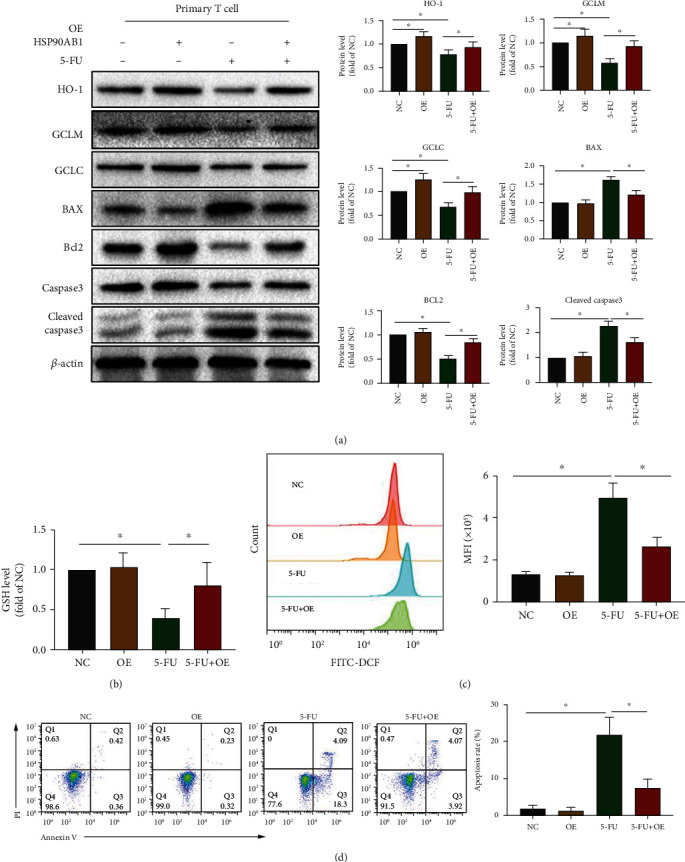
Overexpression of HSP90AB1 can attenuate the inhibition of CAP on GCLC, GCLM, and HO-1 expression and CAP-induced apoptosis in T cells. (a) The protein levels of GCLC, GCLM, HO-1, BCL2, Caspase3, and BAX were evaluated using western blot assay. (b) The GSH level of T cells was evaluated using the GSH Assay Kit. (c) The ROS level of T cells was evaluated using DCFH-DA staining. (d) The apoptosis of T cells was evaluated using Annexin V/PI staining. Data are shown as mean ± SD. ^∗^*P* < 0.05.

Next, 5-FU (the active ingredient of CAP) (10 *μ*M) was chosen to be cultured with T cells for 48 h [12]. Compared with the NC group, HSP90AB1, *p*-HSP90AB1, SMARCC1, *p*-Akt, *p*-c-Fos, *p*-c-Jun, HO-1, GCLC, and GCLM expression levels in the 5-FU group were significantly reduced (Figures 6(a) and 7(a)) and GSH levels were significantly decreased (Figure 7(b)). Compared with the NC group, ROS level (Figure 7(c)) and apoptosis rate in the 5-FU group were significantly increased (Figure 7(d)). Subsequently, compared with the 5-FU group, overexpression of HSP90AB1 in T cells significantly increased SMARCC1, p-Akt, p-c-Fos, p-c-Jun, HO-1, GCLM, and GCLC expression in the 5-FU+OE group (Figures 6(a) and 7(a)). Compared with the 5-FU group, overexpression of HSP90AB1 in T cells increased the GSH level and reduced the ROS level in the 5-FU+OE group (Figures 7(b)–7(d)). The above results confirm that CAP induces T cell apoptosis through the HSP90AB1/Akt/SMARCC1/AP-1/ROS axis.

## 4. Discussion

CAP may be a potential immunosuppressant with an antitumor effect. Studies have also confirmed that CAP can induce T cell apoptosis to exert an immunosuppressive effect [12], but its underlying mechanism is still unclear. For this reason, we chose the metronomic chemotherapy dosage of CAP to treat normal mice by gavage to further explore the mechanism of CAP's immunosuppressive effect.

First, we used quantitative proteomic and phosphoproteomic analyses to comprehensively explore the protein molecular network relevant to the immunosuppressive effect of CAP. Considering the objective difficulties of sorting mouse T cells for proteomic analysis, such as small sample size and many interference factors, we chose the peripheral immune organ—the spleen—for proteomic analysis. CAP can significantly reduce the expression of HSP90AB1 in the spleen of mice. Of course, the spleen houses numerous immune cells [39, 40]. Moreover, changes in the protein content of the spleen are not necessarily consistent with changes in the protein content of T cells. Subsequently, we also confirmed that CAP can significantly reduce HSP90AB1 expression in T cells. As a stress protein, HSP90AB1 is a member of the heat shock protein family [26, 41]. It is also closely related to many physiological functions of cells [28, 42]. Previous studies have confirmed that HSP90AB1 is closely related to T cell activity [25], and other studies have confirmed that HSP90AB1 can regulate the expression of Akt [26]. This study confirmed that knockdown or overexpression of HSP90AB1 in T cells can regulate Akt expression accordingly. Akt is widely involved in T cell activation and proliferation [43, 44]; so, reducing Akt expression in T cells is an important direction for inducing immunosuppressive effects. Sánchez et al. [45] confirmed that targeting the inhibition of Akt expression can further reduce T cell activation and prevent the development of graft-versus-host disease. Chaudhuri et al. [46] confirmed that activating Akt could inhibit T cell apoptosis. This prompted us to hypothesize that HSP90AB1 may be a key target protein for CAP to induce T cell apoptosis. In our study, knockdown of HSP90AB1 in T cells significantly inhibited Akt expression and induced ROS production and apoptosis, which confirmed this hypothesis. It is also worth noting that previous studies have shown that the elevation in intracellular ROS levels can both activate and inhibit Akt signaling [47–50]. Hence, the decrease of Akt expression and the increase of ROS in T cells caused by CAP deserve further study in the future. In addition to HSP90AB1, proteomics results showed that CAP can significantly reduce SMARCC1 expression in the spleen. We also confirmed that CAP can significantly reduce the expression of SMARCC1 in T cells. SMARCC1, which is part of the SWI/SNF complex in the nucleus, is the main complex of ATP-dependent chromatin remodeling factors [51, 52]. Jeong et al. [30] confirmed that in T cells, SMARCC1 is recruited to the promoter of the transcription factor AP-1 and increases AP-1 expression; so, knocking down SMARCC1 reduces AP-1 expression and further regulates T cell activity. Furthermore, previous studies have confirmed that SMARCC1 is the phosphorylation substrate of Akt [31], which led us to believe that HSP90AB1 may regulate SMARCC1 through Akt in T cells. When we knocked down SMARCC1 expression in T cells, there was no significant change in HSP90AB1 or Akt expression. However, when we knocked down the expression of HSP90AB1, the expression of Akt and SMARCC1 decreased significantly. GCLC and GCLM, which were subunits of mammalian glutamate cysteine ligase holoenzyme, can increase GSH level [33, 53]. GSH can regulate the metabolic activity of T cell; in turn, it could also affect T cell activity [53]. HO-1, which is considered to be an antioxidant, can regulate the production of ROS [54]. Several signaling molecules, such as AP-1 and PI3K/Akt, participate in the regulation of HO-1, GCLM, and GCLC expression [33]. In this experiment, as HSP90AB1 or SMARCC1 were knocked down in T cells, AP-1, HO-1, GCLC, and GCLM expression decreased, the GSH level decreased, and the ROS level and apoptosis rate increased. Therefore, we hypothesized that the HSP90AB1/Akt/SMARCC1/AP-1/ROS axis may be the underlying mechanism of CAP-induced T cell apoptosis. T cells and 5-FU (the active component of CAP) were cultured in vitro, and the expression of HSP90AB1, Akt, SMARCC1, AP-1, HO-1, GCLC, and GCLM protein was significantly reduced, the GSH level decreased, and the ROS level and apoptosis rate increased. Overexpression of HSP90AB1 significantly increased the expression of Akt, SMARCC1, AP-1, HO-1, GCLC, and GCLM, increased the GSH level, and reduced the ROS level and apoptosis rate, which confirmed our hypothesis.

## 5. Conclusions

**Figure 8:**
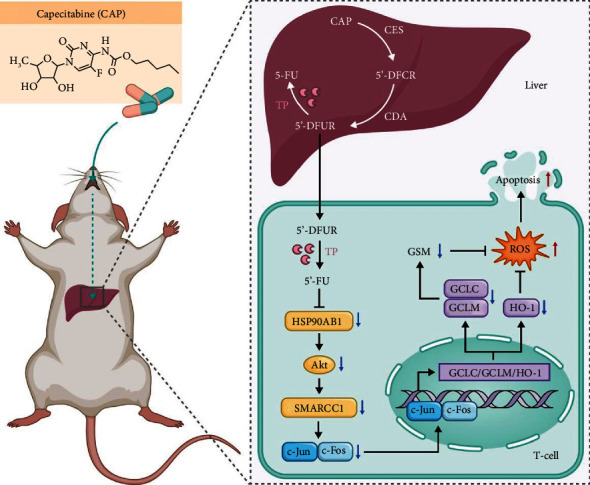
Scheme summarizing the apoptosis of T cells induced by CAP via HSP90AB1/Akt/SMARCC1/AP-1/ROS axis. After oral administration, CAP is converted into 5′DFCR and 5′DFUR by CES and CDA in the liver. In T cells, which expressed TP, 5′DFUR can finally be converted into 5-FU; CAP reduces HSP90AB1, Akt, SMARCC1, c-Fos, c-Jun, GCLC, GCLM, and HO-1 expression, reduces the GSH level, increases the ROS level, and finally induces apoptosis.

In summary, targeting HSP90AB1 is the key to CAP-induced T cell apoptosis, and the HSP90AB1/Akt/SMARCC1/AP-1/ROS axis is the underlying mechanism (Figure 8). Of course, there were some shortcomings in the present study; notably, the activities of antioxidant enzymes such as SOD, CAT, and GPx, which play an important role in eliminating ROS, were not evaluated, and this experiment was based on normal mice and not on organ transplant mice with acute rejection. Therefore, in the future, we will comprehensively explore the mechanism of ROS production and apoptosis induced by CAP in T cells and will not only use an acute rejection mouse model of organ transplantation but also attempt to establish a tumor-bearing animal model simultaneously bearing an organ allograft to confirm that CAP, which targets HSP90AB1 to induce apoptosis, has both immunosuppressive and anticancer effects. In brief, the results of this study provide novel understanding of CAP-induced T cell apoptosis and lay the experimental foundation for further exploring the immunosuppressive effect of CAP to enrich the treatment strategies of transplant oncology and fill the gap of the lack of pyrimidine immunosuppressive agents in the field of organ transplantation.

## Data Availability

The original contributions presented in the study are included in the article/Supplementary Material. The quantitative proteomic and phosphoproteomic data can be found at http://proteomecentral. http://proteomexchange.org/cgi/GetDataset? ID=PX D030193 with the dataset identifier PXD030193. Further inquiries can be directed to the corresponding authors.

## Supplementary Materials

Supplementary Materials Concise supplementary material description: Figure S1: (a–c) GO enrichment analyses based on differentially expressed proteins (DEPs) in quantitative proteomic analysis. Figure S2: (a–c) GO enrichment analyses based on DEPs in phosphoproteomic analysis. Figure S3: (a) the correlation analysis of phosphorylated protein and protein quantification; (b) the Venn diagrams of DEPs in quantitative proteomic and phosphoproteomic analyses on days 7, 14, and 21; (c) protein–protein interaction (PPI) analysis of HSP90AB1, Akt, SMARCC1, c-Fos, c-Jun, GCLC, GCLM, HO-1, BAX, BCL2, and Caspase3.
