# Supplementary material for: Capecitabine Regulates HSP90AB1 Expression and Induces Apoptosis via Akt/SMARCC1/AP-1/ROS Axis in T Cells
Source: Oxid Med Cell Longev. 2022 Mar 24;2022:1012509. doi: 10.1155/2022/1012509 (PMC8970866; doi:10.1155/2022/1012509)
Supplement: Supplementary Materials — Concise supplementary material description: Figure S1: (a–c) GO enrichment analyses based on differentially expressed proteins (DEPs) in quantitative proteomic analysis. Figure S2: (a–c) GO enrichment analyses based on DEPs in phosphoproteomic analysis. Figure S3: (a) the correlation analysis of phosphorylated protein and protein quantification; (b) the Venn diagrams of DEPs in quantitative proteomic and phosphoproteomic analyses on days 7, 14, and 21; (c) protein–protein interaction (PPI) analysis of HSP90AB1, Akt, SMARCC1, c-Fos, c-Jun, GCLC, GCLM, HO-1, BAX, BCL2, and Caspase3. [file 1012509.f1.doc]

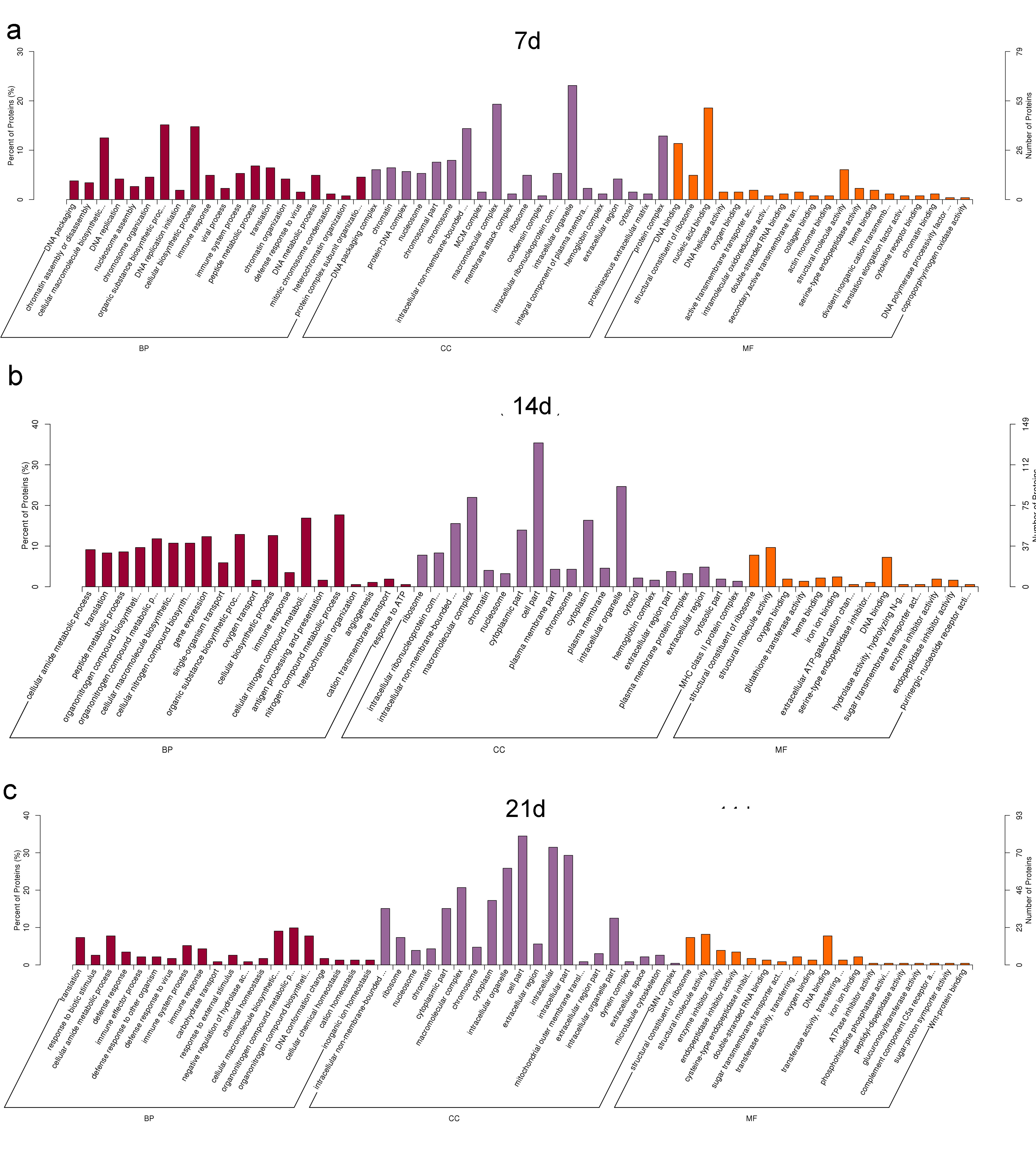


**Figure S1: GO enrichment analyses based on differentially expressed proteins (DEPs) in quantitative proteomic analysis.** Normal mice were administered metronomic doses of CAP (100 mg/kg/d). On days 0, 7, 14, and 21, spleens were collected for quantitative proteomic analysis. Subsequently, GO analysis for DEPs was carried out **(a-c)**.


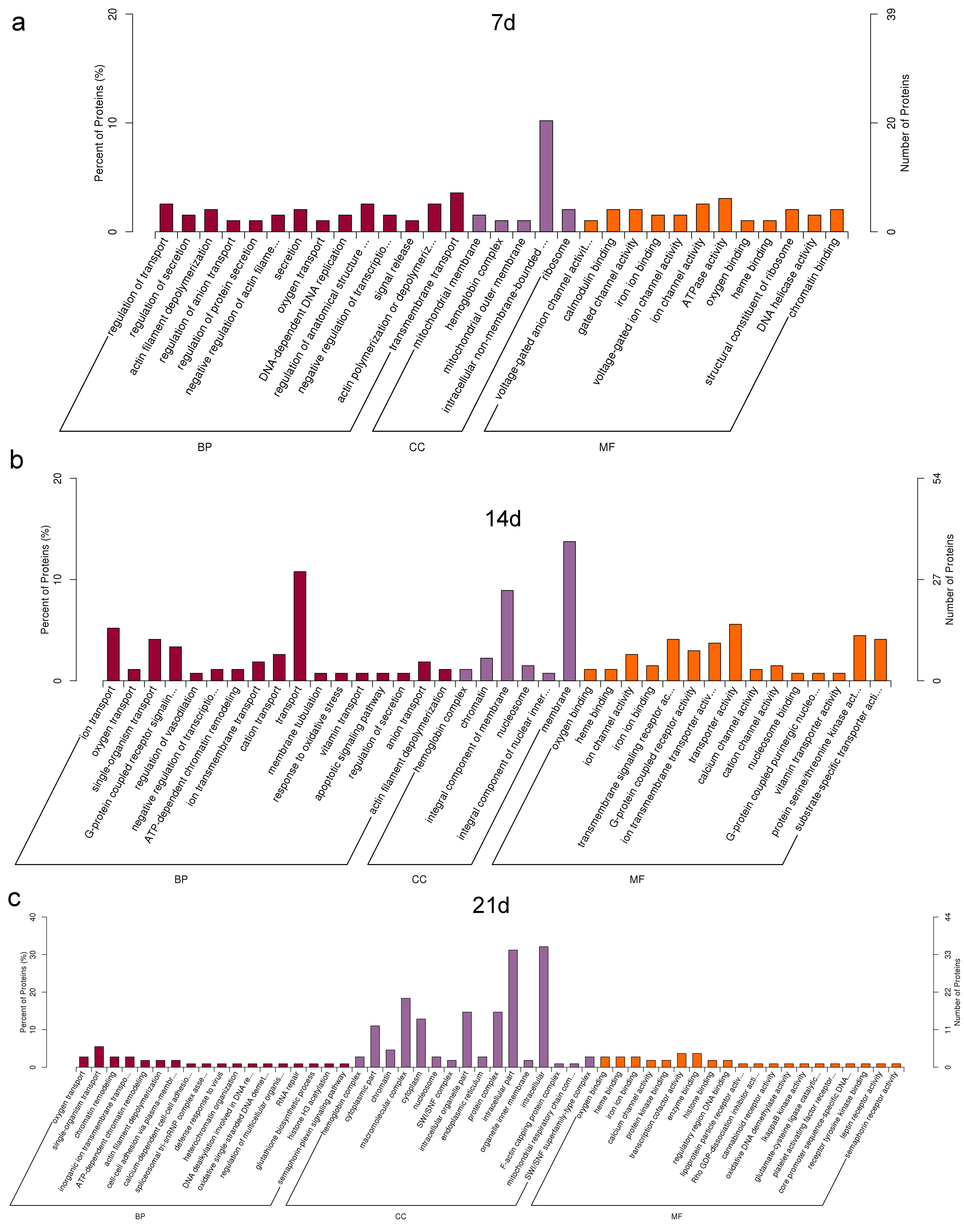


**Figure S2: GO enrichment analyses based on DEPs in phosphoproteomic analysis.** Normal mice were administered metronomic doses of CAP (100 mg/kg/d). On days 0, 7, 14, and 21, spleens were collected for phosphoproteomic analysis, GO analysis for DEPs was carried out **(a-c)**.


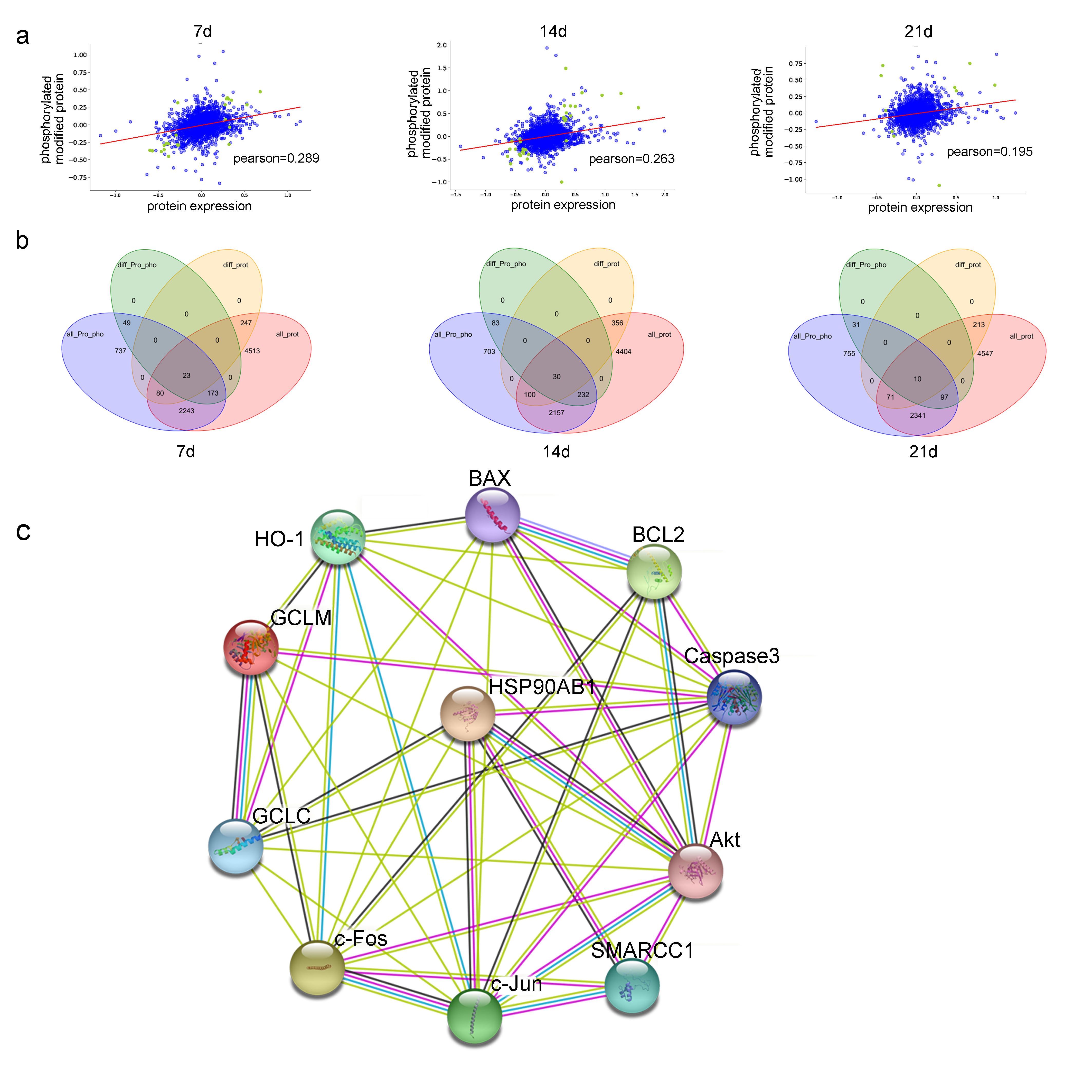


**Figure S3: Association analysis of quantitative proteomic and phosphoproteomic analyses results. (a)** The [correlation analysis](http://www.youdao.com/w/correlation analysis/" \l "keyfrom=E2Ctranslation) of phosphorylated protein and protein quantification; **(b)** The Venn diagrams of DEPs in quantitative proteomic and phosphoproteomic analysis on days 7, 14, and 21; **(c)** Protein–protein interaction (PPI) analysis of HSP90AB1, Akt, SMARCC1, C-Fos, C-Jun, GCLC, GCLM, HO-1, BAX, BCL2 and Caspase3.
